# Retraction: Chikusetsu saponin IVa ameliorates high fat diet-induced inflammation in adipose tissue of mice through inhibition of NLRP3 inflammasome activation and NF-κB signaling

**DOI:** 10.18632/oncotarget.28781

**Published:** 2025-11-14

**Authors:** Chengfu Yuan, Chaoqi Liu, Ting Wang, Yumin He, Zhiyong Zhou, Yaoyan Dun, Haixia Zhao, Dongming Ren, Junjie Wang, Changcheng Zhang, Ding Yuan

**Affiliations:** ^1^College of Medical Science, China Three Gorges University, Yichang, HuBei 443002, China; ^2^Renhe Hospital of China Three Gorges University, Yichang, HuBei 443002, China

**This article has been retracted:** This decision was made following an investigation by Oncotarget into concerns raised by a reader and a request from the corresponding author. An image forensics analysis revealed multiple internal and external duplications, overlaps, and reuse of the Western Blots (WB) images within the article. These issues include.

Internal Duplications:

Figure 4C: The WB of NFkBp65 in epididymal adipose tissue (EAT) is a duplicate of the image of nonspecific bands from NBDM cells in Figure 5D; Figure 4C: IKK alpha bands overlap with alpha-TBP bands in Figure 7C; Figure 4C: The WB of IKK alpha is a duplicate of IkB WB in Figure 7A; Figure 5B: The image of nonspecific bands was reused in Figure 5E as betta-actin bands; Figure 5C: beta actin bands are overlapping with pro-caspase 1 bands in the WB in Figure 5F; Figure 5F: The beta-actin image for BMDM cells is a duplicate of beta-actin bands in Figure 6C/SKOV3 cells; Figure 7A; IkB WB bands overlap with alpha-TBP bands in Figure 7C.

Reuse of Images from a 2016 article [1]:

Figure 4B: The WB for Caspase 1 image was found to overlap with the p-beta-catenin image in Figure 5C [1]; Figure 4B: The beta-actin image is overlapping with HEY cells’ beta-actin image in Figure 6C [1]; Figure 4B: The beta-actin bands are overlapping with beta-catenin bands in Supplementary Figure S2B [1]; Figure 5F: The WB for Caspase 1 was previously used in Figure 5C [1] as GSK 3 beta WB in SKOV3 cells; Figure 5F: The beta-actin image for NBDM cells was a reuse of a previously published beta-actin image for SKOV3 cells in Figure 3D [1].

External Duplication:

We also found that the beta-actin WB in Figure 4B was used in later published, unrelated paper [2] as beta-actin in Figure 3A [2].

Given these findings, the editorial decision has been made to retract the paper. Although the authors initially sent a retraction agreement letter, they did not confirm their agreement with the retraction text.

Original article: Oncotarget. 2017; 8:31023–31040. 31023-31040. https://doi.org/10.18632/oncotarget.16052
